# Misdiagnosed Branchio-Oto-Renal syndrome presenting as proteinuria and renal insufficiency with insidious signs since early childhood: a report of three cases

**DOI:** 10.1186/s12882-023-03193-3

**Published:** 2023-08-23

**Authors:** Zhilang Lin, Jie Li, Yuxin Pei, Ying Mo, Xiaoyun Jiang, Lizhi Chen

**Affiliations:** Department of Pediatric Nephrology and Rheumatology, The First Affiliated Hospital, Sun Yat-sen University, 510080 Guangzhou, P. R. China

**Keywords:** Branchio-oto-renal syndrome, Proteinuria, Renal insufficiency, Kidney failure, Genetic testing, Immune complex-mediated glomerulonephritis, Children

## Abstract

**Background:**

Branchio-oto-renal (BOR) syndrome is an inherited multi-systemic disorder. Auricular and branchial signs are highly suggestive of BOR syndrome but often develop insidiously, leading to a remarkable misdiagnosis rate. Unlike severe morphological abnormalities of kidneys, knowledge of glomerular involvement in BOR syndrome were limited.

**Case presentation:**

Three cases, aged 8 ~ 9 years, visited pediatric nephrology department mainly for proteinuria and renal insufficiency, with 24-h proteinuria of 23.8 ~ 68.9 mg/kg and estimated glomerular filtration rate of 8.9 ~ 36.0 mL/min/1.73m^2^. Moderate-to-severe albuminuria was detected in case 1, while mixed proteinuria was detected in case 2 and 3. Insidious auricular and branchial fistulas were noticed, all developing since early childhood but being neglected previously. *EYA1* variants were confirmed by genetic testing in all cases. Delay in diagnosis was 8 ~ 9 years since extra-renal appearances, and 0 ~ 6 years since renal abnormalities. In case 1, therapy of glucocorticoid and immunosuppressive agents to accompanying immune-complex mediated glomerulonephritis was unsatisfying.

**Conclusions:**

BOR syndrome is a rare cause of proteinuria and abnormal kidney function and easily missed, thus requiring more awareness. Careful medical history taking and physical examination are essential to early diagnosis. Massive proteinuria was occasionally seen in BOR syndrome, which might be related to immune complex deposits. A novel pathogenic variant (NM_000503.6 (*EYA1*): c.1171delT p.Ser391fs*9) was firstly reported.

## Background

Branchio-oto-renal (BOR1, OMIM 113,650; BOR2, OMIM 610,896) syndrome is a rare developmental disorder characterized by hearing loss, preauricular pits, branchial anomalies and renal anomalies [1]. Generally, BOR syndrome is inherited in an autosomal dominant manner, with high penetrance close to 100% but with apparently variable appearances even in a same family [2, 3]. *EYA1* is the main causative gene [4]. Most BOR syndrome cases fulfilled the Chang criteria (2004) [3], which defined major and minor appearances according to clinical manifestations. However, lack of awareness and insidious onset could easily lead to a missed or delayed diagnosis.

The incidence of renal anomalies in BOR syndrome was estimated to be 38 ~ 67% [3, 5], mainly according to data from non-nephrology departments [6]. Knowledge of renal involvement was concentrated in gross morphological abnormalities like renal agenesis, hypoplasia (RH), dysplasia (RD), multicystic dysplastic kidney, polycystic kidney and vesicoureteral reflux [5], however, a proportion of patients presented as glomerular diseases [7–9], which could be misleading in diagnosis and thus results in delayed management. Besides, pathologic data of renal biopsy in BOR syndrome was rather limited [10].

Herein, data of BOR syndrome cases referred to pediatric nephrology department with delayed diagnosis was reported, mainly presenting as various degrees of proteinuria and renal insufficiency. A novel variant in *EYA1* was identified.

## Case presentation

### Case 1

#### Case 1

**Fig. 1:**
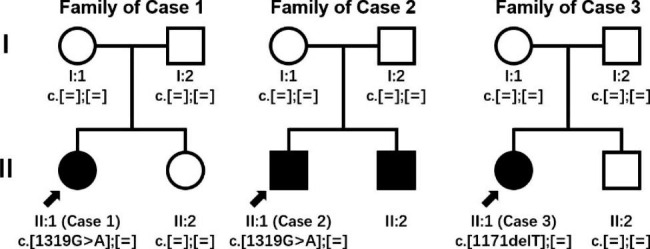
Pedigrees of three BOR syndrome cases. Filled black symbols for males (squares) and females (circles) represent patients fulfilling Chang Criteria. Empty symbols represent unaffected individuals. An arrow denotes the probands. *EYA1* variants by genetic testing using peripheral blood were presented. Except brother of case 2, all underwent Genetic testing

(II:1 in Fig. 1 Family 1) was a 9-year-old female, found to have proteinuria, hypoproteinemia, and renal insufficiency at the age of 8, without edema. Examination at local hospitals revealed nephrotic syndrome and chronic kidney disease (CKD) stage 3. Examinations for immune diseases or other secondary nephrotic syndrome were all negative. Mesangial proliferative glomerulonephritis with immune complex (IC) deposition and segmental sclerosis were diagnosed after renal biopsy. Genetic testing for the first time revealed no variant with sufficient evidence, accompanied with other variants without family verification. As diagnosis of immune-complex mediated glomerulonephritis (ICGN) was considered, methylprednisolone (1 mg/kg/d) and mycophenolate mofetil (MMF, 0.25 g/d) were given. One year later, no significant improvement was observed, accompanied with side-effects including recurrent abdominal pain and potential growth inhibition. No related family history was noted.

**Fig. 2:**
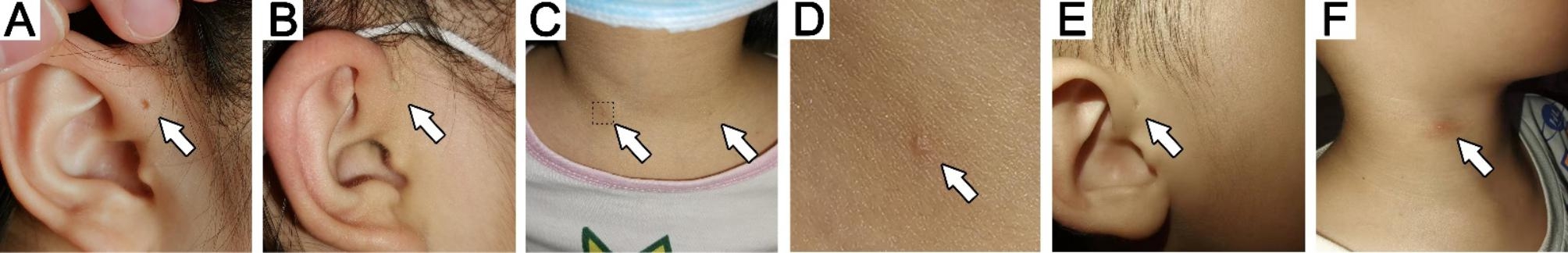
Insidious dysmorphic features. (**A** and **B**) Right preauricular fistula of case 1, with (**B**) or without (**A**) non-purulent secretion. (**C**) Bilateral cervical fistulas of case 1. (**D**) Magnification of the right cervical fistulas of Case 1 (black box in (**C**)), demonstrating its insidious nature. (**E** and **F**) Right preauricular fistula (**E**) and cervical fistula (**F**) of the brother of case 2

**Fig. 3:**
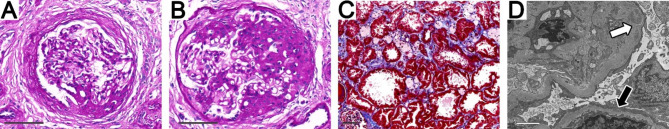
Renal biopsy pathology of Case 1. (**A** and **B**) Glomerular enlargement, segmental sclerosis, mild-to-moderate mesangial proliferation, capsular adhesion, periglomerular fibrosis and arteriole wall thickening. Masson stain, original magnification ×200. Bar 80 μm. (**C**) Foam cells in clusters, interstitial fibrosis, tubular dilatation, and tubular atrophy, without significant inflammatory infiltrates. No pathological changes of polycystic kidney disease. Masson stain, original magnification ×100. Bar 80 μm. (**D**) Electron-dense deposits in subendothelial area, subepithelial area (black arrow), mesangium, and basement membrane (white arrow). Transmission electron microscopy, original magnification ×8000. Bar 2 μm

During physical examination, unexpectedly, bilateral preauricular fistulas and anterior cervical fistulas were found (Fig. 2A, B, C and D). Preauricular fistulas were congenital while anterior cervical fistulas were noticed at the age of 5. Examination (detail in Table 1) revealed proteinuria of 39.0 ~ 68.9 mg/kg/d and renal insufficiency. Tests for autoimmune diseases or infection were negative. Area under the curve of MMF was 47.6 µg.hr/mL (reference range: 20 ~ 60). Ultrasound and magnetic resonance imaging revealed small kidneys with increased echogenicity, reduced number of calyces, and bilateral small renal cysts in cortex. Pure tone audiometry test revealed mild hearing loss. Diagnosis of BOR syndrome was established. Renal biopsy specimens were re-stained and reassessed (Fig. 3). With appropriate sampling, only six glomeruli were observed, revealing glomerular enlargement (231 ~ 252 μm of diameter), segmental glomerular sclerosis, mild-to-moderate proliferation of mesangial cells with partial periglomerular fibrosis, and multifocal tubular atrophy (~ 40%) with foam cells and interstitial fibrosis. Immunofluorescence revealed IgG+, IgM+, C3+, C1q+, depositing focally, segmentally, and granularly. Electron microscopy revealed dense deposition in subendothelial area, mesangium and glomerular basement membrane. By Trio-Whole Exome Sequencing, a heterozygous variant (NM_000503.6 (*EYA1*): c.1319G > A p.Arg440Gln) was corrected as “pathogenic” with added family verification and clinical evidence (PS2 + PS4 + PM2 + PP3), while carrier tests in her parents and sister were negative.

Weighing the pros and cons, MMF treatment was disused and glucocorticoid was reduced. Six months later, follow-up revealed stable level of proteinuria and estimated glomerular filtration rate, without progressing. However, her parents insisted on restarting methylprednisolone therapy at a local clinic. Nevertheless, after 3 months, no relieve of proteinuria was noted, which further reflected the uselessness of immunosuppressive therapy.

#### Case 2

(II:1 in Fig. 1 Family 2) was an 8-year-old boy, with a complaint of increased creatine detected for 2 years. At the age of 2, an operation was performed due to bilateral congenital preauricular fistulas. At the age of 6, bilateral catheterization of tympanic membrane was performed due to secretory otitis media, during which an examination revealed blood urea nitrogen of 9.3 mmol/L and serum creatinine (SCr) of 101 µmol/L. Left RD and bilateral multiple real cysts were detected. CKD stage 3 was diagnosed, with treatment including α-ketoacid tablets and sodium bicarbonate tablets. Inherited disease was suspected but further examination was refused.

At admission, branchial and otic scars were not noticeable, but branchial and preauricular pits of his brother was informed (Fig. 2E and F). Examinations revealed azotemia, hyperuricemia (uric acid of 533.8 µmol/L) and anemia (hemoglobin of 103 g/L). Urinary analysis revealed proteinuria of 31.9 mg/kg/d (detail in Table 1). Ultrasound revealed small kidneys, lacking distinct cortical–medullary demarcation. Pure tone audiometric evaluation revealed moderate conductive hearing loss on the right and mild mixed hearing loss on the left. BOR syndrome was diagnosed. A heterozygous variant (NM_000503.6 (*EYA1*): c.1319G > A p.Arg440Gln) was revealed by genetic testing (PS2 + PS4 + PM2 + PP3 + PP4). His biological parents were excluded to carry the same variant. Oral iron supplementation, calcium supplement and calcitriol were added. Kidney transplant was being waited. After great effort, his medical record at the age of 2 was accessed, revealing SCr of 78 µmol/L (reference: 32.8 ~ 52.0 µmol/L), which was mistakenly labeled as “normal” at that time.

#### Case 3

(II:1 in Fig. 1 Family 3) was an 8-year-old girl with complaints of cough for 2 weeks and increased creatinine detected for 10 days. No history of edema or associated family history was noted. Physical examination revealed a pit at her left mandibular angle and bilateral preauricular pits. Examination revealed CKD stage 5, acidosis (CO_2_CP of 12 mmol/L), anemia (hemoglobin of 93 g/L), and hyperthyroidism (intact parathyroid of 1156 pg/mL). Liver function, albumin level, tests for etiology, tests for rheumatology and autoimmunology were normal. Urinary analysis revealed proteinuria of 23.8 ~ 32.7 mg/kg/d (detail in Table 1). Urinary ultrasound and magnetic resonance imaging revealed size of 6.7 cm × 3.0 cm, multiple cysts, enhanced echogenicity of the parenchyma, lack of distinct cortical–medullary demarcation and lower blood flow on the left kidney; size of 5.8 cm × 3.9 cm and multicystic dysplasia of the right kidney. Pure tone audiometric evaluation revealed moderate to severe hearing impairment. Genetic testing revealed a heterozygous variant (NM_000503.6 (*EYA1*): c.1171delT p.S391fs*9), a novel pathogenic frameshift mutation (PVS1 + PS2 + PM2) without previous report. Her parents and her brother were excluded to carry the same variant. Peritoneal dialysis was conducted while allograft renal transplantation was performed 3 months later. Follow-up at 1 year revealed normal function of transplanted kidney.

**Table 1:** Clinical characteristics of three cases with BOR syndrome in this study^*^

Characteristics	Case 1	Case 2	Case 3
Gender	Female	Male	Female
Age at presentation of renal anomalies (yrs)	8	2	8
Age at presentation of extra-renal anomalies (yrs)	0	0	0
Age at diagnosis (yrs)	9	8	8
Family history	None	Brother	None
Height (cm)	115.5 cm (<-3SD)	122 cm (-2.2SD)	121 cm (-1.5SD)
Weight (kg)	18.7 Kg (-2.6SD)	21 kg (-2.1SD)	23.2 Kg (-1.6SD)
Preauricular pits	Yes	Yes	Yes
Branchial anomalies	Yes	Yes	Yes
Hearing loss	Yes	Yes	Yes
Urine volume (mL/24 h) (mL/kg/24 h)	1850 ~ 2220 (94 ~ 112)	1000 ~ 1700 (48 ~ 81)	700 ~ 1300 (30 ~ 56)
urine specific gravity	1.009 ~ 1.011	1.005 ~ 1.008	1.006 ~ 1.010
urine protein (mg/kg/24 h)	2+ (39.0 ~ 68.9)	±~+ (31.9)	±~+ (23.8 ~ 32.7)
urine ALB mg/L (NRV: 0 ~ 30.00)	996	214	426
urine B2M mg/L (NRV: 0 ~ 0.206)	1.2	11.4	16.2
urine A1M mg/L (NRV: 0 ~ 12.00)	7.0	28.6	45.1
urine IgG mg/L (NRV: 0 ~ 8.5)	26.2	19.6	29.7
urine RBP mg/L (NRV: 0 ~ 0.70)	0.55	6.29	15.86
eGFR (mL/min/1.73m^2^)	36.0	19.4	8.9
SCr (µmol/L)	117	229	495
BUN (mmol/L)	23.0	17.1	31.4
CysC (mg/L)	2.57	2.74	4.47
ALB (g/L)	34	44	44
Chol (mmol/L)	8.5	4.6	4.8
Hb (g/L)	126	103	93
iPTH pg/mL	80.1	56.5	1156.0
K^+^ mmol/L	3.81	3.51	5.37
Imaging	RH (L + R)	RH (L + R)	RH (L + R)
*EYA1* variant (NM_000503.6)	c.1319G > A (p.Arg440Gln), Het	c.1319G > A (p.Arg440Gln), Het	c.1171delT (p.S391fs*9), Het

^*^Data at diagnosis

NRV, normal reference range; ALB, albumin; A1M, α-1-microglobulin; B2M, β-2-microglobulin; BUN, blood urea nitrogen; Chol, cholesterol; CysC, cystatin C; eGFR, evaluated glomerular filtration rate; Hb, hemoglobin; Het, heterozygous; IgG, immunoglobin G; iPTH, intact parathyroid; L, left; r, right; RBP, retinol binding protein; RH, renal hypoplasia; SCr, serum creatinine; yrs, years

## Discussion

We describe three pediatric patients with BOR syndrome, presenting mainly as proteinuria and renal insufficiency with varying degrees. These cases developed BOR syndrome-related appearances in early childhood but the diagnosis was missed until admission in our department. We also reported a novel pathogenic variant of *EYA1*, and infrequent pathological data of renal biopsy in BOR syndrome.

*EYA1*-related BOR syndrome is one of the causes of end-stage kidney disease (ESKD) and kidney transplantation [24]. As an inherited disorder, it is special that adequate awareness of BOR syndrome and clinical presentation are enough for identifying typical cases. The widely used criteria, Chang criteria [3], defined major appearances (branchial anomalies, deafness, preauricular pits and renal anomalies) and minor appearances (external ear anomalies, middle ear anomalies, inner ear anomalies, preauricular tags, and others (facial asymmetry and palate abnormalities)) of BOR syndrome. All our cases could be clinically diagnosed, but the delay in diagnosis was remarkable. It is necessary to enhance awareness of BOR syndrome especially for nephrologists and otolaryngologists. A detailed collection of auricular/branchial history and physical examination is important for those suspected for inherited kidney diseases, but not only focusing on hearing impairment, as in Alport syndrome.

The proportion of renal involvement in BOR syndrome could be underestimated, as systematic renal examination was not conducted in a significant number of patients first visiting non-nephrology departments. Wang et al. [25] indicated an association between auricular malformations and renal anomalies, emphasizing that children with both auricular anomalies and branchial cysts/sinuses require renal ultrasound. We emphasize that renal examination may not only lead to a precise diagnosis, but also promote early multi-organ management and thus delays the progression. Besides, in case 2, mild renal insufficiency arose but was ignored at the age of 2, which was attributed to misjudgment on pediatric SCr levels in departments mainly for adults.

Although BOR syndrome is an autosomal dominant disorder, undiagnosed patients nowadays usually carry *de novo* variants and lack family history. It is worth noting that case 2 and his brother were patients with BOR syndrome but *EYA1* variants were excluded in peripheral blood of their parents, in which the most probable mechanism is germinal mosaicism of *EYA1* in one of their parents, just as Miyagawa’s report [26]. Sporadic cases make it more difficult to recognize this autosomal dominant inherited syndrome. Moreover, as some patients may lack branchial and otic signs, like case 2 who had undergone operations, genetic testing has become an important method for precise diagnosis in BOR syndrome. Nevertheless, the first genetic testing of case 1 did not reveal the true etiology, which was mainly due to inaccurate physical examination, and a lack of family validation. Thus, we highlight the importance of qualified genetic testing and emphasize to improve evidence collection when a strong conclusion has not been achieved.

It was estimated that only 10% of researches on BOR syndrome were published in the field of nephrology [6], leading to a limited understanding of renal involvement. Knowledge of renal injury had been concentrated in morphological abnormalities [5], on the contrary, less is known about glomerular involvement in BOR syndrome. Case 2 and case 3 presented both glomerular and tubular proteinuria, while moderate to severe glomerular proteinuria was detected in case 1, which might be attributed to ICGN. According to reports of proteinuria in BOR syndrome (summarized in Table 2), mild to severe proteinuria could be found, while nephrotic-range proteinuria accounted for around 1/3 ~ 1/4. However, except in our cases, components analysis of proteinuria had never been reported. As tubular involvement is also common in BOR syndrome and easily accompanied with proteinuria, more data is still needed to unveil a more comprehensive picture of the BOR syndrome-related proteinuria. Anyhow, we emphasize that BOR syndrome is one of the rare causes of nephrotic-range proteinuria, especially when accompanied with renal insufficiency, in both children and adults.

**Table 2:** Clinical information of BOR with proteinuria reported in the literature

Year [Ref]	Age-Gender	Familial / Sporadic	Time to diagnosis (yrs)	Gene variant (*EYA1*)	Proteinuria	Hematuria	SCr	CCr/GFR	Imaging
1982 [12]	5-M	Familial	5	-	Slight	No	-	69 mL/min/1.73m^2^ (Cin)	RD (L); RH (R)
1982 [12]	33-M	Familial	33	-	0.5 g/d	-	50	-	RH (R)
1984 [16]	6-F	Familial	6	-	Moderate	-	-	decreased	RA (L); RH (R)
1986 [17]	--M	Familial	-	-	No detail	-	-	ESKD	RH (R)
1986 [17]	19-M	Familial	-	-	No detail	-	-	ESKD	RA (L); RH (R)
1988 [13]	9-F	Familial	-	-	34.2 mg/m^2^/h	No	300	-	RH/RD (L + R)
1989 [18]	39-M	Familial	36	-	4.8 g/d	No	1050	3 mL/min	RH/RD (L + R)
1998 [19]	44-M	Familial	36	-	1.1 g/d	Yes	168	40 mL/min	RH/RD (L + R)
2008 [20]	26-M	Familial	26	c.1376 + 2T > C, Het	4+, ACR 4.9	Yes	1235	-	RH/RD (L + R)
2010 [21]	7-F	Sporadic	7	a 17 kb deletion, Het	0.23 g/d	No	85	55 mL/min/1.73m^2^	RH/RD (L + R)
2011 [22]	23-F	Sporadic	18	1420-1421delCC, Het	3 g/d	No	141	42 mL/min/1.73m^2^	RH/RD (L)
2012 [22]	21-F	Sporadic	-	c.880 C > T, Het	2+	-	97	54 mL/min/1.73m^2^	RH (R)
2013 [7]	27-M	Sporadic	21	c.1475 + 1G > C, Het	2.5 g/d	No	80	131 mL/min	RH (L)
2013 [23]	4(d)-F	Familial	0	-	0.3 g/L	Yes (Mild)	205	10 mL/min/1.73m^2^	RH (L + R)
2016 [9]	16-M	Familial	6	c.1627 C > T, Het	4+, 2.738 g/d	No	633	12 mL/min/1.73m^2^	RH (L + R)
2018 [8]	4-F	Sporadic	3	c.1381delA, Het	2+, 0.44 g/d	No	505	< 15 mL/min/1.73m^2^	RH (L)

Dashes represent data non-available. ACR, albumin-to-creatinine ratio; Cin, inulin clearance; ESKD, end-stage kidney disease; F, female; Het, Heterozygous; L, left; M, male; R, right; RA, renal agenesis; RD, renal dysplasia; RH, renal hypoplasia; yrs, years

Pathology of renal biopsy in BOR syndrome had been only reported in eight cases (summarized in Table 3), in which sclerosis, mesangial proliferation and tubular injury were the common presentations. In our case 1, diffused segmental sclerosis, mesangial deposit and tubular injury were also observed, while electron microscopy revealed dense deposition in subendothelial area, mesangial area and basement membrane. Given the types, intensity and multi-location characteristics of the deposits, it was more like accompanying with ICGN, while the common etiologies of which had been excluded by laboratory examinations. In previous cases of BOR syndrome, IC deposits were occasionally reported, but lacking further details or discussion. Similar to our case, other inherited developmental disorder complicated with ICGN had been occasionally reported, including cohorts of RH [27], *WT1*-nephropathy [28], Alport Syndrome [29], a case of *COL4A5* nephropathy [30], etc. Although the mechanism has not been elucidated, it indicated that those structural renal anomalies may increase the incidence of ICGN. In *EYA1*-related BOR syndrome, *EYA1* variants affect EYA-SIX network and lead to abnormalities of nephron progenitors [8], which may result in congenital anomalies of the kidneys. We speculate the congenital anomalies and secondary disorders (like glomerular hyperfiltration) together result in a higher susceptibility of IC deposits in a proportion of cases with BOR syndrome. The highly variable expression of *EYA1* could explained why only a small proportion of patients presented massive albuminuria. As for treatment, in our case, immunosuppressive therapy to ICGN in this situation seems unsatisfying, but more data is still needed. It is generally believed that kidney transplantation in BOR syndrome has a favorable outcome [8].

**Table 3:** Clinical information and renal pathology of BOR cases reported in the literature

Year [Ref]	Age-Sex	Familial / Sporadic	Renal involvement	Imaging	Main characteristics of renal biopsy pathology
1979 [11]	24-F	Familial	-	RH (R)	Chronic interstitial nephritis.
1982 [12]	25-M	Familial	ESKD	-	Segmental and glomerular sclerosis; mesangial proliferation; mesangial and subendothelial deposits; Atrophied tubules, interstitial fibrosis, and lymphocytes infiltrate.
1982 [12]	33-M	Familial	0.5 g/d of proteinuria; Normal eGFR	RH (R)	Segmental hyalinization; increased mesangial matrix; nodular mesangial deposits; granular deposits of IgM.
1982 [12]	5-M	Familial	Slight proteinuria; Cin of 69 mL/min/1.73m^2^	RH (R + L)	Only 3 glomeruli; segmental and focal hyalinosis with deposits; discrete cellular hyperplasia; subendothelial and focal subepithelial deposits; foamy histiocytes; normal tubules and vessels; Granular deposits of IgM+++, IgA++, C3+, and C4+.
1988 [13]	8-F	Familial	No proteinuria; Normal eGFR	RH (R)	Focal mesangial sclerosis; mesangial proliferation; thickening of the glomerular basement membranes; No deposits of immunoglobulins or complement.
2013 [7]	27-M	Sporadic	2.5 g/d of proteinuria; Normal eGFR	RH (L)	Segmental sclerosis; deposits of IgM and C3.
2015 [14]	35-M	Familial	-	RH (R + L)	Glomerular sclerosis.
2021 [15]	32-M	Sporadic	Normal eGFR	RH (R)	Larger glomerulus (295.4 μm); Focal mesangial proliferation; arteriolosclerosis; focal effacement of the foot process; focal weak granular deposits of IgA and IgM.

Dashes represent data non-available. F, female; M, male; ESKD, end-stage renal disease; Cin, Inulin clearance; RH, renal hypoplasia; RD, renal dysplasia; R, right; L, left

## Conclusion

Awareness of BOR syndrome needs to be improved. Revealing auricular/branchial involvement with careful medical history taking and physical examination is essential for early diagnosis. Genetic testing is an supplementary approach for sporadic cases. Part of patients complicated with various degrees of proteinuria and renal insufficiency, while massive proteinuria could appear occasionally, in which IC deposits might play a role. Moreover, a novel pathogenic variant NM_000503.6 (*EYA1*): c.1171delT p.Ser391fs*9 is firstly reported in this study.

## Data Availability

Detailed data of the cases in this study are available on request from the corresponding author upon reasonable request.

## Abbreviations

BOR: Branchio-oto-renal
CKD: chronic kidney disease
ESKD: end-stage kidney disease
RH: hypoplasia
RD: dysplasia
IC: immune complex
ICGN: immune-complex mediated glomerulonephritis
MMF: mycophenolate mofetil
SCr: serum creatinine
